# Association of Long-Term Trajectories of Neighborhood Socioeconomic Status With Weight Change in Older Adults

**DOI:** 10.1001/jamanetworkopen.2020.36809

**Published:** 2021-02-05

**Authors:** Dong Zhang, Cici Bauer, Tiffany Powell-Wiley, Qian Xiao

**Affiliations:** 1 Department of Family and Preventive Medicine, University of Arkansas for Medical Sciences, Little Rock; 2 Department of Biostatistics and Data Science, School of Public Health, University of Texas Health Science Center at Houston, Houston; 3 National Heart, Lung, and Blood Institute, National Institutes of Health, Bethesda, Maryland; 4 National Institute on Minority Health and Health Disparities, National Institutes of Health, Bethesda, Maryland; 5 Department of Epidemiology, Human Genetics and Environmental Sciences, School of Public Health, University of Texas Health Science Center at Houston, Houston

## Abstract

**Importance:**

Studying long-term changes in neighborhood socioeconomic status (SES) may help to better understand the associations between neighborhood exposure and weight outcomes and provide evidence supporting neighborhood interventions. Little previous research has been done to examine associations between neighborhood SES and weight loss, a risk factor associated with poor health outcomes in the older population.

**Objective:**

To determine whether improvements in neighborhood SES are associated with reduced likelihoods of excessive weight gain and excessive weight loss and whether declines are associated with increased likelihoods of these weight outcomes.

**Design, Study, and Participants:**

This cohort study was conducted using data from the National Institutes of Health-AARP (formerly known as the American Association of Retired Persons) Diet and Health study (1995-2006). The analysis included a cohort of 126 179 adults (aged 50-71 years) whose neighborhoods at baseline (1995-1996) were the same as at follow-up (2004-2006). All analyses were performed from December 2018 through December 2020.

**Exposures:**

Living in a neighborhood that experienced 1 of 8 neighborhood SES trajectories defined based on a national neighborhood SES index created using data from the US Census and American Community Survey. The 8 trajectory groups, in which high, or H, indicated rankings at or above the sample median of a specific year and low, or L, indicated rankings below the median, were HHH (ie, high in 1990 to high in 2000 to high in 2010), or stable high; HLL, or early decline; HHL, or late decline; HLH, or transient decline; LLL, or stable low; LHH, or early improvement; LLH, or late improvement; and LHL, or transient improvement.

**Main Outcomes and Measures:**

Excessive weight gain and loss were defined as gaining or losing 10% or more of baseline weight.

**Results:**

Among 126 179 adults, 76 225 (60.4%) were men and the mean (SD) age was 62.1 (5.3) years. Improvements in neighborhood SES were associated with lower likelihoods of excessive weight gain and weight loss over follow-up, while declines in neighborhood SES were associated with higher likelihoods of excessive weight gain and weight loss. Compared with the stable low group, the risk was significantly reduced for excessive weight gain in the early improvement group (odds ratio [OR], 0.87; 95% CI, 0.79-0.95) and for excessive weight loss in the late improvement group (OR, 0.89; 95% CI, 0.80-1.00). Compared with the stable high group, the risk of excessive weight gain was significantly increased for the early decline group (OR, 1.19; 95% CI, 1.08-1.31) and late decline group (OR, 1.13; 95% CI, 1.04-1.24) and for excessive weight loss in the early decline group (OR, 1.15; 95% CI, 1.02-1.28). The increases in likelihood were greater when the improvement or decline in neighborhood SES occurred early in the study period (ie, 1990-2000) and was substantiated throughout the follow-up (ie, the early decline and early improvement groups). Overall, we found a linear association between changes in neighborhood SES and weight outcomes, in which every 5 percentile decline in neighborhood SES was associated with a 1.2% to 2.4% increase in the risk of excessive weight gain or loss (excessive weight gain: OR, 1.01; 95% CI, 1.00-1.02 for women; OR, 1.02; 95% CI, 1.01-1.03 for men; excessive weight loss: OR, 1.02; 95% CI, 1.01-1.03 for women; OR, 1.02; 95% CI, 1.01-1.03 for men; *P* for- trend < .0001).

**Conclusions and Relevance:**

These findings suggest that changing neighborhood environment was associated with changes in weight status in older adults.

## Key Points

**Question:**

What is the association between neighborhood socioeconomic status (SES) change and weight change among older adults?

**Findings:**

In this cohort study of 126 179 US adults, long-term improvement in neighborhood SES was associated with lower risk for excessive weight gain and excessive weight loss, while long-term neighborhood SES decline was associated with higher risks for these outcomes. There was a dose-dependent association, with larger changes in risk observed with larger neighborhood changes.

**Meaning:**

This study found that sustained neighborhood changes were associated with significant differences in weight outcomes among older adults.

## Teaser

This cohort examines the association of changes in neighborhood socioeconomic status with excessive weight gain and loss among older adults.

## Introduction

Neighborhood environment is a critical factor associated with health outcomes, including obesity.^1,2^ A 2020 systematic review^2^ of longitudinal studies found that declines in measures of neighborhood socioeconomic status (neighborhood SES) were associated with higher weight gain and obesity risk. However, several gaps exist in current literature; to our knowledge, most studies used 1-time snapshots of neighborhood SES and failed to capture neighborhood change, and almost all studies focused on obesity or mean weight change while ignoring weight loss, a unique risk factor associated with morbidity and mortality in the older population.^3^

Neighborhoods change over time. Thus, characterizing long-term neighborhood trajectories is crucial for assessing the cumulative and changing exposure to environmental factors among individuals in those neighborhoods. Moreover, because it is challenging to conduct large experiments examining changing neighborhood conditions, studying improvements, declines, and fluctuations that naturally occur in the neighborhood offers an alternative approach for understanding the association between neighborhood exposure and health outcomes and providing meaningful information for developing future interventions. Only a few studies, to our knowledge, have examined the association of changes in neighborhood SES with weight outcomes in adults, and these studies reported mixed findings: 2 studies^4,5^ found that neighborhood improvements were associated with reduced weight gain and a lower risk for obesity, a study by Powell-Wiley et al^6^ found that declines in neighborhood SES were associated with increased weight gain, and 2 studies^7,8^ reported null findings. The inconsistency in previous studies warrants further investigations of neighborhood change and weight outcomes.

Although moderate weight loss in overweight populations is beneficial, an evolving literature has consistently found that excessive weight loss is associated with health decline and increased mortality, particularly in the older population.^9,10,11,12^ Neighborhood changes may influence dietary options, social interactions, and health status of residents, all of which have been associated with unintentional weight loss.^13^ Although studies from 2011,^14^ 2006,^15^ and 2002^16^ examined the association of neighborhood SES with weight loss in adults, none of these studies focused on changes in neighborhood SES as an exposure.

In a large cohort of older adults, we examined the association of trajectories of neighborhood SES between 1990 and 2010 with weight change over 10 years of follow-up. We hypothesized that improvements in neighborhood SES would be associated with lower likelihoods of excessive weight gain and weight loss while declines would be associated with higher likelihoods of these weight-associated outcomes.

## Methods

### Study Population

The National Institutes of Health-AARP (formerly known as the American Association of Retired Persons) Diet and Health Study was established in 1995 by recruiting AARP members aged 50 to 71 years from 6 US states (ie, California, Florida, Louisiana, New Jersey, North Carolina, and Pennsylvania) and 2 metropolitan areas (ie, Atlanta, Georgia, and Detroit, Michigan).^17^ The study was approved by the National Cancer Institute Special Studies Institutional Review Board. All participants gave informed consent by virtue of completing and returning the questionnaire. The Strengthening the Reporting of Observational Studies in Epidemiology (STROBE) reporting guideline was followed.

At baseline, 556 388 participants completed a self-administered survey reporting height, weight, and residential addresses, along with sociodemographic variables, lifestyle factors, and health status. In 2004 to 2005, a follow-up survey was mailed to study participants using their most up-to-date mailing addresses. A total of 318 713 study participants returned the follow-up survey, which collected updated information on weight. For this analysis, we focused on 169 223 nonmovers, defined as participants whose 2004 to 2005 address was within 1 km of their baseline address. Of these, we further excluded participants who did not report weight, had extreme body mass index (BMI; calculated as weight in kilograms divided by height in meters squared) levels (ie, <15 or >50) at baseline (4051 individuals) or follow-up (25 428 individuals), had no neighborhood SES information (6258 individuals), or had an address that could not be matched to an exact street or point address (7307 individuals). The final analytic cohort included 76 225 men and 49 954 women. Study characteristics according to inclusion status are presented in eTable 1 in the Supplement, and the 2 groups are largely comparable.

### Neighborhood SES Trajectories

We derived a national neighborhood SES index for all US census tracts for 1990, 2000, and 2010 separately using census data (from the 1990, 2000, and 2010 censuses) and the 2006 to 2010 American Community Survey. Based on the procedure developed by Messer et al,^18^ we selected 14 variables (eTable 2 in the Supplement) associated with different domains of the neighborhood environment and performed principal component analysis (PCA) for 1990, 2000, and 2010 separately. The first PCA component was used to generate year-specific national percentile rankings for all census tracts. Next, we created 8 trajectory groups, in which high, or H, indicated rankings at or above the sample median of a specific year and low, or L, indicated rankings below the median: HHH (ie, high in 1990 to high in 2000 to high 2010), or stable high; HLL, or early decline; HHL, or late decline; HLH, or transient decline; LLL, or stable low; LHH, or early improvement; LLH, or late improvement; and LHL, or transient improvement.

To assess the dose-dependent association, we derived a continuous variable of neighborhood change by subtracting the 1990 ranking from the 2010 ranking. Because this measure captures only the difference and not the fluctuation between 1990 and 2010, we excluded 2350 neighborhoods (17.1%) with substantial SES fluctuation, defined as having changes in ranking of 5 percentile points or more in the 1990 to 2000 period and 2000 to 2010 period, with changes in ranking from the 2 time periods in opposite directions.

### Weight Change

Excessive weight gain or loss were defined as gaining or losing 10% or more of baseline weight. Studies from 2001,^19^ 2019,^20^ and 2002^21^ found associations between weight change and health in older adults using various cutoff points (4%-10% of baseline weight) over varied follow-up durations (5-12 years). We chose 10% over 10 years of follow-up because it represented a substantial deviation from the baseline weight. Moreover, using this criterion, reasonably large proportions of the study population were defined as having excessive weight gain (14 264 individuals [11.3%]) and loss (9624 individuals [7.6%]), allowing for sufficient statistical power for the analysis.

### Statistical Analysis

We used multinomial logistic regression models to calculate odds ratios (OR) and 95% CIs. The outcome variable included 3 categories: gaining 10% or more of baseline weight, losing 10% or more of baseline weight, and gaining or losing less than 10% of baseline weight (ie, the reference group). We conducted separate analyses for the initially advantaged neighborhoods (ie, HHH, HLL, HHL, and HLH groups) and initially disadvantaged neighborhoods (ie, LLL, LHH, LLH, and LHL groups). In each analysis, the stable group (ie, HHH or LLL groups) served as the reference.

We considered a series of models. Model 1 was adjusted for age and sex. Model 2 was additionally adjusted for race/ethnicity and education. In model 3 (the main model), we further included neighborhood SES in 1990. In model 4, we additionally adjusted for lifestyle factors (ie, physical activity, dietary quality, smoking status, and alcohol consumption status) and self-rated health, which are likely mediators of the association between neighborhood SES and weight outcomes. In all models, we used robust variance estimation to account for clustering of participants in census tracts. Finally, we conducted restricted cubic splines analysis to examine the dose-dependent association between changes in neighborhood SES and weight outcomes.^22,23,24^ Because results from the spline models indicated a significant linear association, we calculated the OR and 95% CI for every 5-percentile change in neighborhood SES.

Hypothesis testing was 2-sided with a significance level of α < .05. All analyses were performed using SAS statistical software version 9.4 (SAS Institute) from December 2018 through December 2020.

## Results

Among 126 179 individuals, 76 225 (60.4%) were men and the mean (SD) age was 62.1 (5.3) years; 116 967 (92.7%) were White, and 52 196 (41.1%) had a college education or higher. In eTable 3 in the Supplement, we presented the median and interquartile range (IQR) of year-specific national neighborhood SES ranking and percentage of households below poverty for all 8 trajectory groups. In this cohort, neighborhood SES rankings ranged from top 0 to bottom 99.9 percentiles, with a median (IQR) of 25.1 (10.8-46.3) for 1990, 28.8 (12.4-52.0) for 2000, and 30.1 for 2010 (12.2-55.2). Compared with individuals in the stable high group (ie, HHH), those in the decline groups (ie, HLL, or early decline; HHL, or late decline; and HLH, or transient decline) had lower mean neighborhood SES and higher mean poverty level in 1990. Conversely, when compared with individuals in the stable low group (ie, LLL), those in the improvement groups (ie, LHH, or early improvement; LLH, or late improvement; and LHL, or transient improvement) had a higher mean neighborhood SES and had a lower mean poverty level in 1990.

Baseline participant characteristics according to trajectory groups are presented in Table 1. Most participants lived in stable neighborhoods (50 076 individuals [39.6%] in the HHH group and 46 136 individuals [36.6%] in the LLL groups). Participants in the decline groups, compared with those in the HHH group, were more likely to be women and report current smoking, but less likely to have a college education, be married, or report excellent health. Participants in the improvement groups, compared with participants in the LLL group, were more likely to be men, married and White; have a college education; and report excellent health. They were less likely to be current smokers or have diabetes at baseline and had higher mean (SD) alcohol consumption.

**Table 1.** Baseline Characteristics by Trajectory of Neighborhood Socioeconomic Status

Characteristic, No (%)	Trajectory of neighborhood SES^a^							
	HHH	HLL	HHL	HLH	LLL	LHH	LLH	LHL
Total	50 076 (39.6)	5696 (4.5)	5817 (4.6)	3442 (2.7)	46 136 (36.6)	5360 (4.3)	5801 (4.6)	3851 (3.1)
Age, mean (SD), y	61.9 (5.3)	62.0 (5.2)	61.9 (5.3)	62.3 (5.3)	62.2 (5.3)	62.4 (5.2)	62.6 (5.2)	62.4 (5.3)
BMI, mean (SD)	26.6 (4.2)	26.9 (4.4)	27.0 (4.5)	26.6 (4.4)	27.4 (4.8)	26.9 (4.3)	26.8 (4.5)	27.2 (4.7)
15 to <25	19 228 (38.4)	2076 (36.5)	2072 (35.6)	1328 (38.6)	15 371 (33.3)	1932 (36.0)	2130 (36.7)	1309 (34.0)
25 to <30	21 886 (43.7)	2406 (42.2)	2505 (43.1)	1476 (42.9)	19 643 (42.6)	2373 (44.3)	2486 (42.9)	1675 (43.5)
30 to <35	6842 (13.7)	925 (16.2)	942 (16.2)	470 (13.7)	7864 (17.1)	792 (14.8)	887 (15.3)	623 (16.2)
35 to 50	2120 (4.2)	289 (5.1)	298 (5.1)	168 (4.9)	3258 (7.1)	263 (4.9)	298 (5.1)	244 (6.3)
Women	17 538 (35.0)	2417 (42.4)	2241 (38.5)	1438 (41.8)	20 552 (44.6)	1954 (36.5)	2330 (40.2)	1484 (38.5)
White, non-Hispanic^b^	47 642 (95.1)	5273 (92.6)	5546 (95.3)	3207 (93.2)	39 823 (86.3)	5149 (96.1)	5371 (92.6)	3671 (95.3)
College and postcollege^b^	26 205 (52.3)	2314 (40.6)	2335 (40.1)	1555 (45.2)	14 253 (30.9)	2077 (38.8)	2196 (37.9)	1261 (32.7)
Married^b^	39 223 (78.3)	3928 (69.0)	4250 (73.1)	2458 (71.4)	30 755 (66.7)	4090 (76.3)	4228 (72.9)	2810 (73.0)
Current smoker^b^	3670 (7.3)	577 (10.1)	556 (9.6)	319 (9.3)	5182 (11.2)	458 (8.5)	519 (9.0)	377 (9.8)
Vigorous physical activity ≥5 times/wk^b^	10 441 (20.9)	1115 (19.6)	1161 (20.0)	709 (20.6)	9023 (19.6)	1127 (21)	1303 (22.5)	800 (20.8)
Alcohol intake, mean (SD), g/d	13.6 (33.1)	12.0 (31.8)	11.6 (31.2)	13.5 (38.2)	11.5 (36.1)	13.5 (36.7)	13.5 (36.5)	13.0 (36.5)
Total energy, mean (SD), kcal/d	1848 (810)	1817 (842)	1832 (797)	1793 (826)	1897 (1007)	1865 (822)	1861 (858)	1906 (954)
HEI-2005 score, mean (SD)	67.7 (10.9)	67.5 (11.0)	67.2 (11.0)	68.2 (11.0)	66.5 (11.5)	67.0 (11.0)	67.2 (11.1)	66.4 (11.3)
Self-reported health, excellent^b^	10 579 (21.1)	998 (17.5)	1081 (18.6)	707 (20.5)	6819 (14.8)	948 (17.7)	1042 (18.0)	584 (15.2)
Chronic conditions^b^								
Heart disease	5773 (11.5)	699 (12.3)	738 (12.7)	369 (10.7)	5612 (12.2)	659 (12.3)	678 (11.7)	495 (12.9)
Stroke	621 (1.2)	64 (1.1)	74 (1.3)	46 (1.3)	800 (1.7)	72 (1.3)	78 (1.3)	61 (1.6)
Diabetes	2947 (5.9)	438 (7.7)	414 (7.1)	209 (6.1)	4005 (8.7)	364 (6.8)	406 (7.0)	297 (7.7)
Cancer	3844 (7.7)	448 (7.9)	422 (7.3)	291 (8.5)	3518 (7.6)	437 (8.2)	501 (8.6)	297 (7.7)

Abbreviations: BMI, body mass index (calculated as weight in kilograms divided by height in meters squared); HHH, high in 1990 to high in 2000 to high in 2010, or stable high; HLL, high-low-low, or early decline; HHL, high-high-low, or late decline; HLH, high-low-high, or transient decline; HEI, healthy eating index; LLL, low-low-low, or stable low; LHH, low-high-high, or early improvement; LLH, low-low-high, or late improvement; LHL, low-high-low, or transient improvement; SES, socioeconomic status.

^a^ Trajectories of neighborhood SES were defined based on the median values of year-specific rankings, where high, or H, indicated rankings at or above the median and low, or L, indicated rankings below the median.

^b^ Percentages are within the neighborhood SES trajectory group.

The associations between neighborhood SES trajectories and excessive weight gain are presented in Table 2. Compared with the HHH group, the risk of excessive weight gain was increased by 19% for the HLL group (OR, 1.19; 95% CI, 1.08-1.31) and 13% for the HHL group (OR, 1.13; 95% CI, 1.04-1.24), after adjusting for potential confounders (model 3). The HLH exhibited a nonsignificant increase in risk of excessive weight gain (OR, 1.06; 95% CI, 0.95-1.19). Compared with the LLL group, the risk of excessive weight gain was lower for the LHH group (OR, 0.87; 95% CI, 0.79-0.95), LLH group (OR, 0.93; 95% CI, 0.85-1.01), and LHL group (OR, 0.92; 95% CI, 0.83-1.03), although the results for the latter 2 groups were not statistically significant. Additional adjustment for lifestyle factors and health status attenuated the association (model 4). Gender-specific results were similar, although LHH was associated with decreased risk of excessive weight gain in men (OR, 0.76; 95% CI, 0.66-0.88) but not women (OR, 0.99; 95% CI, 0.87-1.13).

**Table 2.** Trajectory of Neighborhood Socioeconomic Status and Excessive Weight Gain^a^

	Trajectory of neighborhood SES, OR (95% CI)^b^							
	Neighborhood SES in 1990 ≥ sample median				Neighborhood SES in 1990 < sample median			
	HHH	HLL	HHL	HLH	LLL	LHH	LLH	LHL
**Overall**								
No. (%)	4952 (9.9)	704 (12.4)	683 (11.7)	375 (10.9)	5912 (12.8)	557 (10.4)	648 (11.2)	433 (11.2)
Model 1^c^	1 [Reference]	1.26 (1.16-1.37)	1.20 (1.10-1.30)	1.10 (0.99-1.23)	1 [Reference]	0.83 (0.76-0.91)	0.89 (0.82-0.97)	0.90 (0.81-1.00)
Model 2^d^	1[Reference]	1.22 (1.12-1.33)	1.16 (1.06-1.26)	1.09 (0.97-1.21)	1 [Reference]	0.85 (0.77-0.93)	0.91 (0.83-0.99)	0.90 (0.81-1.00)
Model 3^e^	1 [Reference]	1.19 (1.08-1.31)	1.13 (1.04-1.24)	1.06 (0.95-1.19)	1 [Reference]	0.87 (0.79-0.95)	0.93 (0.85-1.01)	0.92 (0.83-1.03)
Model 4^f^	1 [Reference]	1.16 (1.05-1.27)	1.11 (1.01-1.21)	1.06 (0.94-1.19)	1 [Reference]	0.89 (0.81-0.98)	0.95 (0.87-1.04)	0.93 (0.83-1.04)
**Women**								
No. (%)	2541 (14.5)	404 (16.7)	369 (16.5)	216 (15.0)	3418 (16.6)	310 (15.9)	358 (15.4)	237 (16.0)
Model 1^c^	1 [Reference]	1.22 (1.09-1.37)	1.18 (1.05-1.32)	1.08 (0.93-1.26)	1 [Reference]	0.95 (0.84-1.08)	0.91 (0.81-1.03)	0.94 (0.81-1.10)
Model 2^d^	1 [Reference]	1.20 (1.07-1.35)	1.15 (1.02-1.29)	1.07 (0.92-1.25)	1 [Reference]	0.95 (0.84-1.08)	0.91 (0.81-1.03)	0.94 (0.80-1.09)
Model 3^e^	1 [Reference]	1.21 (1.06-1.38)	1.16 (1.02-1.31)	1.08 (0.92-1.27)	1 [Reference]	0.99 (0.87-1.13)	0.94 (0.83-1.07)	0.98 (0.83-1.14)
Model 4^f^	1 [Reference]	1.18 (1.04-1.35)	1.14 (1.00-1.29)	1.07 (0.92-1.26)	1 [Reference]	1.01 (0.89-1.16)	0.96 (0.84-1.09)	0.98 (0.83-1.15)
**Men**								
No. (%)	2411 (7.4)	300 (9.2)	314 (8.8)	159 (7.9)	2494 (9.8)	247 (7.3)	290 (8.4)	196 (8.3)
Model 1^c^	1 [Reference]	1.30 (1.15-1.48)	1.22 (1.07-1.38)	1.13 (0.96-1.33)	1 [Reference]	0.72 (0.63-0.83)	0.87 (0.77-0.98)	0.85 (0.73-0.99)
Model 2^d^	1 [Reference]	1.25 (1.10-1.42)	1.16 (1.02-1.31)	1.10 (0.94-1.30)	1 [Reference]	0.75 (0.65-0.86)	0.90 (0.80-1.02)	0.86 (0.75-0.99)
Model 3^e^	1 [Reference]	1.17 (1.02-1.35)	1.11 (0.97-1.26)	1.05 (0.88-1.24)	1 [Reference]	0.76 (0.66-0.88)	0.91 (0.80-1.03)	0.87 (0.74-1.02)
Model 4^f^	1 [Reference]	1.13 (0.98-1.31)	1.06 (0.93-1.21)	1.04 (0.88-1.23)	1 [Reference]	0.78 (0.67-0.91)	0.94 (0.82-1.06)	0.89 (0.75-1.04)

Abbreviations: HHH, high in 1990 to high in 2000 to high in 2010, or stable high; HLL, high-low-low, or early decline; HHL, high-high-low, or late decline; HLH, high-low-high, or transient decline; LLL, low-low-low, or stable low; LHH, low-high-high, or early improvement; LLH, low-low-high, or late improvement; LHL, low-high-low, or transient improvement; OR, odds ratio; SES, socioeconomic status.

^a^ Excessive weight gain was defined as gaining 10% or more of baseline body weight.

^b^ Trajectories of neighborhood SES were defined based on the median values of year-specific rankings, where H indicated rankings at or above the median and L indicated rankings below the median.

^c^ Model 1 adjusted for age (continuous) and sex (ie, men and women, for overall analysis alone).

^d^ Model 2 adjusted for variables in model 1, race/ethnicity (ie, White, Black, or other), and education (ie, <12 y, high school graduate, some college, college and higher).

^e^ Model 3 adjusted for variables in model 2 and neighborhood SES ranking in 1990 (continuous).

^f^ Model 4 adjusted for variables in model 3 and physical activity (ie, never, rarely, 1-3 times/mo, 1-2 times/wk, 3-4 times/wk, or ≥5 times/wk), Healthy Eating Index score (continuous), smoking status (ie, current, former, or never), alcohol consumption status (ie, nondrinker, <2 drinks/wk, 2 drinks/wk to 1 drink/d, or ≥1 drink/d), and self-rated health (ie, excellent, very good, good, fair, or poor).

The associations between neighborhood SES trajectories and excessive weight loss are presented in Table 3. Overall, a decline in neighborhood SES was associated with a higher likelihood of excessive weight loss, while an improvement in neighborhood SES was associated with a lower likelihood of excessive weight loss. Specifically, compared with the HHH group, the HLL group had a significantly increased likelihood of excessive weight loss (OR, 1.15; 95% CI, 1.02-1.28) (model 3), although the increases for the HHL group (OR, 1.03; 95% CI, 0.91-1.15) and HLH group (OR, 1.08; 95% CI, 0.95-1.23) were not significant. We also observed an approximate 10% reduction in the risk of excessive weight loss for the LHH group (OR, 0.91; 95% CI, 0.80-1.02) and LLH group (OR, 0.89; 95% CI, 0.80-1.00) compared with the LLL group. Analyses in men and women separately did not reveal significant differences. Adjusting for lifestyle factors and health status (ie, model 4) was associated with attenuation of the associations but not with a change in the main patterns of the results.

**Table 3.** Trajectory of Neighborhood Socioeconomic Status and Excessive Weight Loss^a^

	Trajectory of neighborhood SES, OR (95% CI)^b^							
	Baseline neighborhood SES ≥ sample median				Baseline neighborhood SES < sample median			
	HHH	HLL	HHL	HLH	LLL	LHH	LLH	LHL
**Overall**								
No. (%)	3331 (6.7)	466 (8.2)	421 (7.2)	267 (7.8)	3982 (8.6)	403 (7.5)	438 (7.6)	316 (8.2)
Model 1^c^	1 [Reference]	1.26 (1.14-1.40)	1.11 (1.00-1.24)	1.16 (1.02-1.32)	1 [Reference]	0.85 (0.76-0.95)	0.85 (0.76-0.94)	0.94 (0.84-1.06)
Model 2^d^	1 [Reference]	1.25 (1.13-1.38)	1.09 (0.98-1.22)	1.16 (1.02-1.31)	1 [Reference]	0.85 (0.76-0.95)	0.85 (0.77-0.95)	0.94 (0.84-1.06)
Model 3^e^	1 [Reference]	1.15 (1.02-1.28)	1.03 (0.91-1.15)	1.08 (0.95-1.23)	1 [Reference]	0.91 (0.80-1.02)	0.89 (0.80-1.00)	1.00 (0.88-1.13)
Model 4^f^	1 [Reference]	1.12 (1.00-1.26)	1.00 (0.89-1.13)	1.10 (0.97-1.26)	1 [Reference]	0.94 (0.83-1.05)	0.93 (0.83-1.03)	1.00 (0.88-1.13)
**Women**								
No. (%)	1287 (7.3)	211 (8.7)	171 (7.6)	116 (8.1)	1984 (9.7)	176 (9.0)	199 (8.5)	132 (8.9)
Model 1^c^	1 [Reference]	1.24 (1.06-1.45)	1.07 (0.90-1.27)	1.11 (0.91-1.35)	1 [Reference]	0.91 (0.77-1.07)	0.85 (0.72-1.00)	0.90 (0.75-1.09)
Model 2^d^	1 [Reference]	1.23 (1.05-1.44)	1.05 (0.89-1.25)	1.11 (0.91-1.35)	1 [Reference]	0.90 (0.76-1.07)	0.85 (0.72-1.00)	0.90 (0.74-1.09)
Model 3^e^	1 [Reference]	1.16 (0.98-1.38)	1.01 (0.84-1.21)	1.06 (0.86-1.30)	1 [Reference]	0.97 (0.81-1.15)	0.89 (0.75-1.06)	0.96 (0.79-1.17)
Model 4^f^	1 [Reference]	1.14 (0.96-1.36)	0.99 (0.82-1.18)	1.08 (0.88-1.33)	1 [Reference]	1.01 (0.84-1.21)	0.93 (0.78-1.10)	0.97 (0.79-1.18)
**Men**								
No. (%)	2044 (6.3)	255 (7.8)	250 (7.0)	151 (7.5)	2494 (7.8)	239 (6.7)	268 (7.1)	189 (7.7)
Model 1^c^	1 [Reference]	1.28 (1.12-1.46)	1.14 (0.99-1.31)	1.21 (1.02-1.43)	1 [Reference]	0.81 (0.70-0.94)	0.84 (0.73-0.97)	0.97 (0.83-1.13)
Model 2^d^	1 [Reference]	1.26 (1.10-1.44)	1.11 (0.97-1.28)	1.19 (1.01-1.42)	1 [Reference]	0.82 (0.71-0.96)	0.86 (0.75-0.99)	0.97 (0.84-1.13)
Model 3^e^	1 [Reference]	1.13 (0.98-1.32)	1.03 (0.89-1.20)	1.10 (0.92-1.32)	1 [Reference]	0.87 (0.74-1.02)	0.90 (0.78-1.03)	1.03 (0.87-1.21)
Model 4^f^	1 [Reference]	1.11 (0.96-1.29)	1.01 (0.88-1.17)	1.12 (0.94-1.34)	1 [Reference]	0.90 (0.77-1.05)	0.93 (0.81-1.07)	1.02 (0.87-1.20)

Abbreviations: HHH, high in 1990 to high in 2000 to high in 2010, or stable high; HLL, high-low-low, or early decline; HHL, high-high-low, or late decline; HLH, high-low-high, or transient decline; LLL, low-low-low, or stable low; LHH, low-high-high, or early improvement; LLH, low-low-high, or late improvement; LHL, low-high-low, or transient improvement; OR, odds ratio; SES, socioeconomic status.

^a^ Excessive weight loss was defined as losing 10% or more of baseline body weight.

^b^ Trajectories of neighborhood SES were defined based on the median values of year-specific rankings, where H indicated rankings at or above the median and L indicated rankings below the median.

^c^ Model 1 adjusted for age (continuous) and sex (ie, men and women, for overall analysis alone).

^d^ Model 2 adjusted for variables in model 1, race/ethnicity (ie, White, Black, or other), and education (ie, <12 y, high school graduate, some college, college and higher).

^e^ Model 3 adjusted for variables in model 2 and neighborhood SES ranking in 1990 (continuous).

^f^ Model 4 adjusted for variables in model 3 and physical activity (ie, never, rarely, 1-3 times/mo, 1-2 times/wk, 3-4 times/wk, or ≥5times/wk), Healthy Eating Index score (continuous), smoking status (ie, current, former, or never), alcohol consumption status (ie, nondrinker, <2 drinks/wk, 2 drinks/wk to 1 drink/d, or ≥1 drink/d), and self-rated health (ie, excellent, very good, good, fair, or poor).

**Figure 1.**
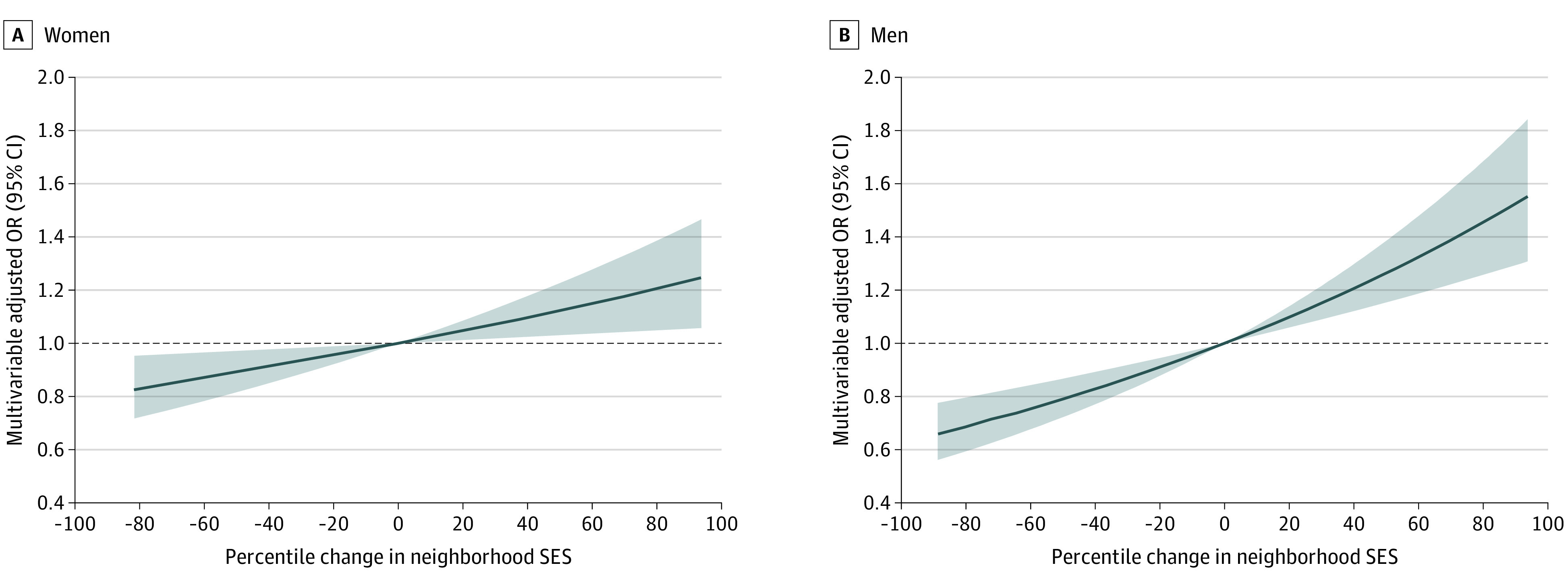
Dose-Dependent Association Between Changes in Neighborhood Socioeconomic Status (SES) and Excessive Weight Gain Without Substantial Fluctuation in Neighborhood SES Substantial fluctuation in neighborhood SES was defined as experiencing changes in ranking of 5 percentile points or more in the 1990 to 2000 period and 2000 to 2010 period, with changes within the 2 periods in opposite directions. Models were adjusted for age (continuous), race/ethnicity (ie, White, Black, or other), education (ie, <12 years, high school graduate, some college, or college and higher), neighborhood SES ranking in 1990 (continuous), physical activity (ie, never, rarely, 1-3 times/mo, 1-2 times/week, 3-4 times/week, ≥5 times/week), Healthy Eating Index score (continuous), smoking status (ie, current, former, or never), alcohol consumption status (ie, nondrinker, <2 drinks/wk, 2 drinks/wk to 1 drink/d, or ≥1 drink/d), and self-rated health (ie, excellent, very good, good, fair, poor). Black lines indicate odds ratios (ORs); shaded areas, 95% CIs.

**Figure 2.**
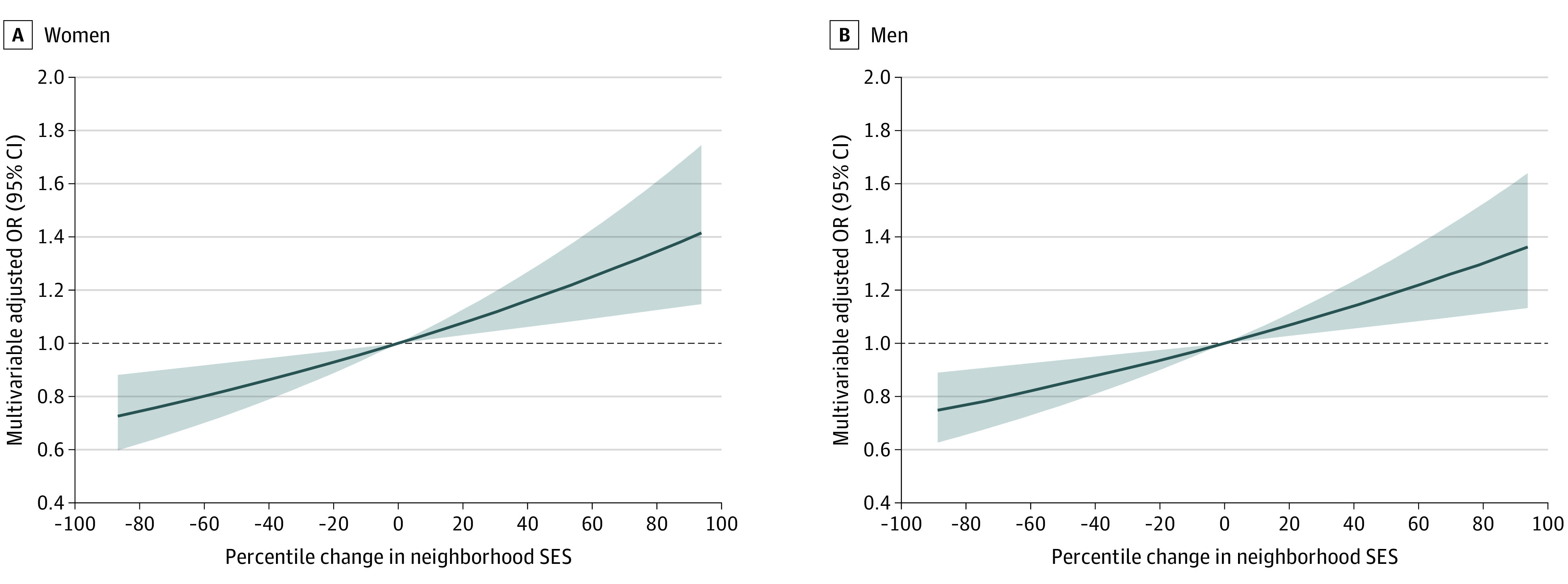
Dose-Dependent Association Between Changes in Neighborhood Socioeconomic Status (SES) and Excessive Weight Loss Without Substantial Fluctuation in Neighborhood SES Substantial fluctuation in neighborhood SES was defined as experiencing changes in ranking of 5 percentile points or more in the 1990 to 2000 period and 2000 to 2010 period, with the changes within the 2 periods in opposite directions. Models were adjusted for age (continuous), race/ethnicity (ie, White, Black, or other), education (ie, <12 years, high school graduate, some college, or college and higher), neighborhood SES ranking in 1990 (continuous), physical activity (ie, never, rarely, 1-3 times/mo, 1-2 times/wk, 3-4 times/wk, or ≥5 times/wk), Healthy Eating Index score (continuous), smoking status (ie, current, former, or never), alcohol consumption status (ie, nondrinker, <2 drinks/wk, 2 drinks/wk to 1 drink/d, or ≥1 drink/d), and self-rated health (ie, excellent, very good, good, fair, or poor). Black lines indicate odds ratios (ORs); shaded areas, 95% CIs.

Finally, we examined the dose-dependent association between changes in neighborhood SES and excessive weight gain (Figure 1) and loss (Figure 2) among individuals living in neighborhoods that did not experience substantial fluctuations in SES from 1990 to 2010. We found a significant linear association between neighborhood SES change and weight gain and loss (*P* for trend < .0001) for men and women. Every 5-percentile decline in neighborhood SES was associated with 1.2% to 2.4% increase in the risk of excessive weight gain or loss (excessive weight gain: OR, 1.01; 95% CI, 1.00-1.02 for women; OR, 1.02; 95% CI, 1.01-1.03 for men; excessive weight loss: OR, 1.02; 95% CI, 1.01-1.03 for women; OR, 1.02; 95% CI, 1.01-1.03 for men).

## Discussion

In this large cohort study of older US adults, we found that, consistent with our hypothesis, participants in neighborhoods with declines in SES were at higher risk of excessive weight gain and loss, while those in neighborhoods with improvements in SES were at lower risk of these outcomes. Moreover, our results showed dose-dependent associations, in which larger improvements and declines were associated with larger differences in risk of adverse weight outcomes.

Several previous investigations on changes in neighborhood SES and weight outcomes reported findings similar to ours. In the Dallas Heart Study (DHS), a population-based cohort study in Dallas County, Texas, Powell-Wiley et al^6^ reported that moving to more disadvantaged neighborhoods was associated with larger weight gain over 7 years of follow up compared with moving to similar or more advantaged neighborhoods. In another DHS study, Leonard et al^4^ characterized neighborhood SES using property appraisal values and found that a 1-SD improvement in neighborhood conditions was associated with 0.7 kg less weight gain, and the association appeared stronger among nonmovers than movers. Additionally, a longitudinal analysis^5^ among California mothers found that moving to a census tract with a lower poverty level was associated with a 50% reduction in the odds of obesity. Overall, these findings and ours suggest that improvements in neighborhood conditions were associated with lower obesity, while residents in deteriorating neighborhoods may be at higher risk for obesity and related chronic conditions.

However, not all study results were consistent with ours. An early investigation in the Multi-Ethnic Study of Atherosclerosis^7^ used latent growth curve models to estimate six 20-year trajectory groups (1980-1999) of neighborhood poverty patterns and found that the trajectory showing substantial reductions in poverty (4.1% of study population) was not associated with BMI. In another study, Kimbro et al^8^ examined the likelihood of obesity in association with within-individual changes in neighborhood conditions and had null findings. Although it is unclear what specific factors may lead to inconsistent results among these studies, all studies, including ours, differed in a number of ways, including population sociodemographic characteristics, geographic regions, measures of neighborhood SES and weight outcomes used, and statistical model characteristics, including controlling of confounders. We need future studies, including original investigations, meta-analyses, and systematic reviews, to clarify the association between changes in neighborhood SES and weight outcomes, identify population and contextual factors that may modulate the associations, and examine methodological issues that may be associated with changes in the results.

A main distinction between our study and the earlier studies was that we treated weight gain and weight loss as separate outcomes. Weight loss is prevalent among older populations; it has been estimated that 15% to 20% of adults aged 65 years or older experienced a 5% or greater reduction in body weight over a relatively short period of time (ie, 6 months to 1 year), often without an intention to lose weight.^13^ Unintentional weight loss has been associated with social isolation, poor nutrition, and chronic diseases, such as cancer, gastrointestinal problems, and mental disorders.^13^ The high prevalence and distinct underlying mechanisms of unintentional weight loss suggest that it should be treated as a unique weight outcome in older populations. Neighborhood environment has been associated with risks for cancer and mental disorders^25,26^ and is a critical factor associated with shaping social interactions, diet, and physical activity behaviors.^27^ Indeed, we found that neighborhood declines were associated with a higher risk for excessive weight loss. However, our observational study was not designed to establish causality, and we did not examine the underlying mechanisms of the observed associations. Future studies should focus on pinpointing the specific pathways through which neighborhood environment may affect weight loss. It has been estimated that weight loss was associated with a 22% to 39% increase in mortality risk in healthy older adults and those with chronic conditions.^12^ Thus, our study results suggest that clinicians and public health officials should pay close attention to weight loss among older adults who live in a neighborhood with declining SES. Moreover, as most of the current research efforts, to our knowledge, focus on obesity, weight loss remains an understudied area and more research is needed to identify modifiable risk factors at the individual and neighborhood levels to inform clinical practices and public health interventions.

Our study measured neighborhood SES at 3 time points, which allowed us to distinguish among changes that occurred early, late, or transiently during the 20-year study period. In most cases, we found that improvements or declines that occurred early tended to be associated with larger increases in risk, suggesting that there may be a lag period for the association of weight with changes in neighborhood SES. Furthermore, the results also indicated that it may require sustained neighborhood changes for a significant association with changes in weight distribution among residents to appear, a potentially important consideration when designing programs aimed at improving neighborhood conditions to promote healthy weight status.

Our study has important strengths, including a large sample size, geographically diverse neighborhoods, and a long follow-up period. Neighborhoods tend to be stable over time. Therefore, it requires a large and diverse population to capture the small fraction of neighborhoods with substantial changes. Another strength of this study is its use of national rankings to assess neighborhood SES, instead of relying on sample-specific measures. This strategy may have reduced the impact of events and trends that are highly specific to the study population. For example, a study that included neighborhoods that, as a whole, experienced deteriorating conditions would characterize a stable neighborhood in this study as an improved neighborhood; the same neighborhood would be characterized as a declined neighborhood in a study that included neighborhoods with largely upward changes in SES. As a result, it may be difficult to generalize the findings from 1 study to others or to the entire country, and the use of national rankings in our current study was associated with reductions in this problem.

### Limitations

This study has several limitations. First, our neighborhood assessments were restricted to the 3 time points when the US Census and ACS were conducted (ie, 1990, 2000, and 2010), while weight status was measured from 1995 to 1996 and 2004 to 2006. The difference in the time frame of exposure and outcome measurements may lead to misclassification, as the actual neighborhood changes may have occurred before 1995 or after 2006. In addition, although we restricted our analysis to individuals who reported living in the same area at both baseline and follow-up, we were not able to identify those who moved out of and back into the baseline neighborhood, which may also lead to exposure misclassification. Also, weight status was reported only at baseline and 10 years later, at follow-up, which did not allow us to assess short-term weight fluctuations. Importantly, gaining or losing weight over a short period of time (ie, several months to years) may be associated with a larger change in health outcomes compared with gradual change in weight over years, and more studies are needed to investigate the association between neighborhood environment and short-term weight change. Additionally, participants in our study were predominantly White and had high SES, as measured by college education or higher; therefore, the results may not be generalizable to other racial/ethnic groups and low SES populations, for whom the association between neighborhood SES and weight may differ from that observed among our participants. The relatively high baseline neighborhood SES has limited our ability to assess the potential association between neighborhood improvement and weight change among residents of disadvantaged communities.

## Conclusions

This cohort study found that changes in neighborhood conditions were associated with excessive weight gain and loss. These findings contribute to the increasing evidence for an association between neighborhood disadvantage and unhealthy weight change. Our results suggest the importance of developing sustainable neighborhood interventions to address health disparities.

## Notes

Supplement. **eTable 1.** Baseline Study Characteristics by Inclusion Status **eTable 2.** Census Variables and Their Principal Component Analysis Loadings Used to Derive Neighborhood Socioeconomic Status Index **eTable 3.** Neighborhood Socioeconomic Status (SES) According to Trajectories of Neighborhood SES
