# Supplementary material for: Association of Long-Term Trajectories of Neighborhood Socioeconomic Status With Weight Change in Older Adults
Source: JAMA Netw Open. 2021 Feb 5;4(2):e2036809. doi: 10.1001/jamanetworkopen.2020.36809 (PMC7865190; doi:10.1001/jamanetworkopen.2020.36809)
Supplement: Supplement. — eTable 1. Baseline Study Characteristics by Inclusion Status eTable 2. Census Variables and Their Principal Component Analysis Loadings Used to Derive Neighborhood Socioeconomic Status Index eTable 3. Neighborhood Socioeconomic Status (SES) According to Trajectories of Neighborhood SES [file jamanetwopen-e2036809-s001.pdf]

Supplemental Online Content

Zhang D, Bauer C, Powell-Wiley T, Xiao Q. Association of long-term trajectories of neighborhood socioeconomic status with weight change in older adults. *JAMA Netw Open*. 2021;4(2):e2036809. doi:10.1001/jamanetworkopen.2020.36809

eTable 1. Baseline Study Characteristics by Inclusion Status

eTable 2. Census Variables and Their Principal Component Analysis Loadings Used to Derive Neighborhood Socioeconomic Status Index

eTable 3. Neighborhood Socioeconomic Status (SES) According to Trajectories of Neighborhood SES

This supplemental material has been provided by the authors to give readers additional information about their work.

© 2021 Zhang D et al. *JAMA Network Open.*

eTable 1. Baseline Study Characteristics by Inclusion Status

	Inclusion Status	
	**Excluded**	**Included**
N (%)	440,219 (77.7)	126,179 (22.3)
nSES 1990, percentile, mean (SD)	33.7 (25.6)	30.9 (24.2)
nSES 2000, percentile, mean (SD)	37.4 (26.6)	34.1 (25.3)
nSES 2010, percentile, mean (SD)	39.7 (27.3)	35.4 (26.5)
Age, year, mean (SD)	62.2 (5.4)	62.1 (5.3)
BMI, kg/m^2, mean (SD)	27.2 (5.3)	26.9 (4.5)
BMI, kg/m^2, % [b]		
15-<25	34.1	36.0
25-<30	44.1	43.2
30-<35	15.4	15.3
35-50	6.4	5.5
Female, %	40.2	39.6
White, non-Hispanic, % [b]	91.1	91.7
College and post-college, % [b]	37.2	41.4
Married, % [b]	67.7	72.7
Current smoker, % [b]	12.8	9.2
Vigorous physical activity≥5 times/week, % [b]	18.7	20.4
Alcohol intake, g/d, mean (SD)	13.4 (39.3)	12.6 (34.7)
Total energy, kcal/d, mean (SD)	1881.7 (1031.7)	1865.5 (895.8)
HEI-2005 score, mean (SD)	66.4 (11.6)	67.2 (11.2)
Self-reported health, excellent, % [b]	15.8	18.0
Chronic conditions, % [b]		
Heart disease	14.8	11.9
Stroke	2.5	1.4
Diabetes	9.9	7.2
Cancer	9.3	7.7

Abbreviations: BMI, body mass index; HEI, healthy eating index; SD, standard deviation; nSES, neighborhood socioeconomic status.

© 2021 Zhang D et al. *JAMA Network Open.*

eTable 2. Census Variables and Their Principal Component Analysis Loadings Used to Derive Neighborhood Socioeconomic Status Index

**Census variables**	**1990 loading**	**2000 loading**	**2010 loading**
Percent of total with less than high school	0.296129	0.311938	0.286432
Percent of total unemployed	0.334534	0.339309	0.303133
Percent of households with income below poverty	0.352683	0.368370	0.357449
Percent of households below a certain income threshold [a]	0.322207	0.346062	0.346014
Percent of households on public assistance	0.351932	0.332701	0.359891
Percent of households with no car	0.303555	0.298681	0.265652
Percent of unemployed men	0.314157	0.318468	0.266748
Percent of renter occupied housing units	0.222267	0.160996	0.267548
Percent of housing units vacant	0.086242	0.114345	0.104945
Median value of all owner-occupied housing units	-0.165036	-0.167387	-0.163699
Percent of female headed households with dependent children	0.322570	0.310668	0.327423
Percent of non-Hispanic blacks.	0.263079	0.240636	0.241336
Percent of residents 65 years and over	0.000869	-0.051791	-0.094652
Percent of persons in same residence since 5 years before the census	-0.012425	-0.081673	-0.153378

[a] The income threshold was set at $22500, $30000, and $40,000 for 1990, 2000 and 2010, repsectively.
Abbreviations: PCA, principle component analysis; SES, socioeconomic index.

© 2021 Zhang D et al. *JAMA Network Open.*

eTable 3. Neighborhood Socioeconomic Status (SES) According to Trajectories of Neighborhood SES

	Overall	Trajectories of Neighborhood SES [a]							
		HHH	HLL	HHL	HLH	LLL	LHH	LLH	LHL
**nSES national ranking, percentile, median (IQR)**									
1990	25.1 (10.8, 46.3)	8.6 (4.0, 14.6)	20.9 (17.3, 24.2)	17.6 (12.7, 21.7)	18.7 (14.6, 22.4)	53.0 (40.2, 69.5)	31.9 (28.5, 37.2)	37.4 (31.1, 46.9)	33.2 (28.7, 38.5)
2000	28.8 (12.4, 52.0)	9.8 (4.5, 16.6)	39.4 (34.3, 46.6)	21.8 (17.0, 25.8)	35.0 (32.2, 40.1)	59.1 (45.8, 74.3)	23.3 (17.9, 26.6)	39.7 (34.4, 48.1)	25.9 (23.2, 28.2)
2010	30.1 (12.2, 55.2)	10.0 (4.3, 17.4)	46.3 (38.5, 56.3)	38.5 (33.9, 46.2)	22.4 (16.0, 26.7)	61.6 (48.4, 77.0)	19.8 (13.7, 25.9)	24.2 (19.2, 28.3)	41.1 (35.3, 49.9)
**% households under poverty, median (IQR)**									
1990	5.6% (3.3%, 9.3%)	3.2% (2.1%, 4.4%)	4.6% (3.6%, 6.0%)	4.0% (2.8%, 5.1%)	4.9% (3.7%, 6.1%)	10.5% (7.6%, 15.3%)	6.9% (5.6%, 8.7%)	8.1% (6.4%, 10.6%)	6.7% (5.0%, 8.5%)
2000	6.2% (3.7%, 10.2%)	3.5% (2.3%, 4.9%)	7.2% (5.6%, 9.4%)	4.7% (3.6%, 6.1%)	7.0% (5.3%, 8.6%)	8.6% (8.6%, 16.5%)	5.3% (4.1%, 6.7%)	8.1% (6.3%, 10.2%)	5.8% (4.5%, 6.8%)
2010	5.0% (2.0%, 9.0%)	2.0% (1.0%, 4.0%)	7.0% (5.0%, 10.0%)	6.0% (4.0%, 8.0%)	3.0% (2.0%, 6.0%)	11.0% (7.0%, 17.0%)	4.0% (2.0%, 5.0%)	4.0% (2.0%, 6.0%)	6.0% (4.0%, 9.0%)

[a]Trajectories of neighborhood SES were defined based on the median values of year-specific rankings, where high, or H, indicated rankings at or above the median and low, or L, indicated rankings below the median. Specifically, the 8 trajectory groups include HHH (ie, high in 1990 to high in 2000 to high 2010), or stable high; HLL, or early decline; HHL, or late decline; HLH, or transient decline; LLL, or stable low; LHH, or early improvement; LLH, or late improvement; and LHL, or transient improvement.

Abbreviations: IQR, interquartile range; nSES, neighborhood socioeconomic status.

© 2021 Zhang D et al. *JAMA Network Open.*
